# Supplementary material for: Dehydroepiandrostenedione sulphate (DHEAS) levels predict high risk of rheumatoid arthritis (RA) in subclinical hypothyroidism
Source: PLoS One. 2021 Feb 16;16(2):e0246195. doi: 10.1371/journal.pone.0246195 (PMC7886134; doi:10.1371/journal.pone.0246195)
Supplement: S1 File — (DOCX) [file pone.0246195.s001.docx]

Supplementary material 1: The EULAR-CSA score

History taking:

- Joint symptoms of recent onset (duration <1 year)
- Symptoms located in MCP joints
- Duration of morning stiffness ≥60 min
- Most severe symptoms present in the early morning
- Presence of a first-degree relative with RA

Physical examination:

- Difficulty with making a fist
- Positive squeeze test of MCP joints

EULAR, European League Against Rheumatism; RA, rheumatoid arthritis
